# Current Endoscopic Treatment of Dysphonia

**DOI:** 10.1155/DTE.6.87

**Published:** 2000

**Authors:** John M. Schweinfurth, Robert H. Ossoff

**Affiliations:** Department of Otolaryngology Vanderbilt University MCN S-2100 Nashville TN 37232-2559 USA

## Abstract

Benign laryngeal disorders result in dysphonia because of effects on glottic closure and the vibratory characteristics of the true vocal fold. Treatment is initially directed at reversing medical conditions and patterns of abuse with surgery reserved for unresolving lesions resulting in troublesome dysphonia. Benign lesions that require surgery are excised as precisely as possible sparing overlying mucosa and the underlying vocal ligament. Vocal fold scarring is currently best treated by augmentation procedures, and atrophy may be compensated for by medialization thyroplasty or by adding bulk to the affected folds. Application of current knowledge of laryngeal histology and physiology is prerequisite to endoscopic surgical intervention.


*Diagnostic and Therapeutic Endoscopy*, Vol. 6, pp. 87–90
Reprints available directly from the publisher
Photocopying permitted by license only

© 2000 OPA (Overseas Publishers Association) N.V.
Published by license under
the Harwood Academic Publishers imprint,
part of The Gordon and Breach Publishing Group.
Printed in Malaysia.

### Current Endoscopic Treatment of Dysphonia

JOHN M. SCHWEINFURTH* and ROBERT H. OSSOFF

*Department of Otolaryngology, Vanderbilt University, MCN S-2100, Nashville, TN 37232-2559, USA*

*(Received 30 July 1999; Revised 20 September 1999; In final form 1 October 1999)*

**Benign laryngeal disorders result in dysphonia because of effects on glottic closure and the vibratory characteristics of the true vocal fold. Treatment is initially directed at reversing medical conditions and patterns of abuse with surgery reserved for unresolving lesions resulting in troublesome dysphonia. Benign lesions that require surgery are excised as precisely as possible sparing overlying mucosa and the underlying vocal ligament. Vocal fold scarring is currently best treated by augmentation procedures, and atrophy may be compensated for by medialization thyroplasty or by adding bulk to the affected folds. Application of current knowledge of laryngeal histology and physiology is prerequisite to endoscopic surgical intervention.**

*Keywords:* Benign, Endoscopic, Larynx, Microflap, Scarring, Surgery

### INTRODUCTION

Videostroboscopy has greatly improved our ability to recognize and diagnose benign laryngeal conditions. Application of the histological model developed by Hirano, Gray and others in recent years has allowed for more specific surgical approaches [1,2]. The vocal fold is composed of a muscle covered by a free mucosal edge which vibrates and can be separated into discrete layers based on the concentration of elastin and collagen fibers which run parallel to the leading edge [2,3]. Histologically, the vocal fold is a complex structure. The delicate arrangement of the extracellular matrix proteins within the lamina propria permits passive movement of the vocal cover over the body resulting in the formation of the mucosal wave as air is passed through the glottis as a release of building subglottic pressure. Most benign lesions occur in the superficial layer of the lamina propria, and therefore surgical approaches to benign lesions should ideally be confined to this layer [4]. Injury to the deeper layers of the lamina propria and the vocal ligament itself can lead to scar formation.

Repeated trauma from vocal misuse or overuse may lead to the development of nodules, polyps, or cysts [5,6]. As mentioned previously, benign lesions are found within the lamina propria and cause dysphonia by disrupting the vibratory pattern and approximation of the true vocal folds. Surgery is reserved for lesions that show no reversibility with exhaustive medical and speech therapy. Known

* Corresponding author. Tel.: (615)322-7267. Fax: (615)343-7604.

sources of mechanical trauma are maximally reduced prior to considering surgical therapy to determine reversibility and hopefully prevent a postoperative recurrence.

### The Microflap

The goal of surgical excision is preservation of the mucosal cover while avoiding injury to other structures. Fibroblasts producing structural tissue components are located in the deeper layers of the lamina propria. Injury to this layer can lead to scarring along the vocal ligament and traction on the mucosal cover. The microflap approach to the excision of benign laryngeal lesions was designed to prevent such an injury [7,8]. The operative technique involves incising the mucosal cover of the vocal fold lateral to the pathology (lateral microflap) or overlying it (medical microflap). The mucosa is then elevated over the lesion, the lesion dissected from the mucosa and underlying vocal ligament, and the mucosal flap replaced. The lateral microflap is selected when the lesion is adherent to the vocal ligament but the overlying mucosa is normal [9]. The incision and subsequent scar remain lateral to the vibratory surface and dissection may proceed from known to unknown beginning in a normal area of vocal fold. In a review of 30 patients undergoing the lateral microflap, Courey *et al.* [9] demonstrated improved glottic closure in 27 of 29 patients with impaired preoperative closure, return of mucosal wave in 18 of 24, and subjective clinical improvement in 28. In six of six patients a preoperative mucosal wave was preserved.

The medial microflap is indicated for lesions that involve a discrete portion of the vocal fold and appear to separate easily from the underlying vocal ligament on palpation [10]. This technique requires less flap elevation and can be useful in cases of redundant or adherent overlying mucosa. An injectable steroid solution may be deposited beneath the flap to reduce scar formation. In a review of 17 patients undergoing the medial microflap procedure for benign pathology, 15 had improved glottic closure and return of mucosal wave [10].

### Varices

Varices and capillary ectasias are most commonly found in female professional vocalists but also occur in men [11]. Current theories attribute the cause to shearing stress along the lateral vocal fold near the termination point of the mucosal wave [12]. Microvascular lesions may be asymptomatic or present secondary to hemorrhage and edema, scarring, or mass effect with resultant disrupution of mucosal wave. Surgery is recommended for recurrent hemorrhage, enlargement of the lesion, development of an associated mass, or intolerable dysphonia. Endoscopic treatment begins by identifying the afferent and efferent vessels which are then respectively photocoagulated with the appropriate laser. Scar formation is minimized with power settings of 1–2 W, pulse width 0.1 s, and spot size of 3–400 μm [12]. The primary lesion may then be excised via a microflap approach or also photocoagulated depending upon its size. Despite appropriate measures there remains the potential for scarring in these patients and conservative management is preferred in the presence of good vocal function.

### Vocal Scarring and Atrophy

The treatment of vocal fold scarring is currently evolving. Non-surgical causes include untreated benign lesions, chronic vocal abuse, and repeated intracordal hemorrhage. Iatrogenic causes include post-surgical dehiscence of the vocal fold mucosa which heals by secondary intention and violation of the vocal ligament and deep layers of the lamina propria. Scarring produces dysphonia by impairing glottic closure and mucosal wave amplitude. This decreased pliability restricts the Bernoulli and myoelastic effects, increasing the effort needed to initiate speech. Yanagihara attributed the resulting harsh vocal quality to an increase in fundamental frequency and significantly reduced harmonics [13].

Dense scar and fibrotic bands should be removed in an atraumatic fashion, preferably through a microflap approach [16]. Research efforts in this area have been directed at the development of a biocompatible material which can be placed between the vocal ligament and cover or within the layers of the lamina propria and potentially function in the place of lost tissue. Ideally this would restore the sliding action of the mucosal cover. Gray *et al.* postulated that this implanted material may also prevent fibroblast migration and further scar formation [14].

Implant material should approximate the function of the intermediate layer of the lamina propria, which is composed of elastin, hyaluronic acid, and fibromodulin [15]. It should augment both the superior and inferior aspects of the free edge of the vocal fold. Because phonation threshold pressure is decreased by improved adduction, increased fold thickness, and lower viscous damping [16] the ideal implant should also have a low viscosity. Abdominal fat is the closest material available to lamina propria in viscosity (4 Pa s) whereas collagen is much higher (10 Pa s) [17]. Autologous fat is therefore probably the best augmentation material currently in widespread use. It has the advantage of positional stability within the vocal fold and longevity through the development of viable adipocytes over time. In an animal model, Archer and Banks demonstrated maintenance of viable adipocytes and bulk for up to one year following implantation [18].

Endoscopic or transcutaneous injection is the most convenient method of implant delivery, but anecdotal reports suggest that sufficient bulk cannot be obtained through injection alone, that passage through a needle barrel is too traumatic to the adipocytes, and that they may simply extrude out of the injection site. On the other hand, implanted fat tends to migrate superiorly in the pocket [18]. Our current technique is to harvest fat via a large 8 mm liposuction cannula and inject it into the bulk of the thyroarytenoid muscle endoscopically. We also follow anecdotal reports which suggest rinsing harvested fat in insulin to support adipocyte cell membrane stabilization. Initial over-medialization should be planned to compensate for postoperative decrease in volume of approximately 30%.

Atrophy of the true vocal folds is most commonly associated with paralysis but occurs with surgery, neurological disorders, and the aging process. Like scarring, atrophy prevents efficient vocal fold closure resulting in breathiness, poor projection, and vocal fatigue. Unlike scarring, the body-cover relationship is not directly affected and mucosal wave is usually present but may be decreased. Phonation threshold pressure is abnormally elevated secondary to an increased glottal width and a decreased fold thickness [16]. The most directed treatment is therefore to add bulk and medialize the leading edge of the vocal fold. Autologous fat implantation is approached as previously described, although medialization thyroplasty has also been used with success [15]. Future injectable materials may more closely simulate the composition of the intermediate layer of the lamina propria but the difficulty remains as to consistent placement and long term positional stability.

### CONCLUSION

Although great strides have been made in the understanding of vocal anatomy, physiology, and pathology, much remains to be understood. Certainly, there is no consensus among surgeons as to how best approach benign laryngeal conditions and a variety of approaches flourish. Scar formation within the vocal cover remains the greatest obstacle both in the development of new operative approaches and the resolution of past injury. The development of finer instrumentation and more precise control along with implantable biomaterials which more closely resemble the original anatomy will determine the future direction of our subspecialty. Regardless, it will remain critical to prevent vocal injury as much as possible by relying on maximum medical therapy and good vocal hygiene.

### *References*

[1] Hirano, M. Structure of the vocal fold in normal and disease states. Anatomical and physical study. *ASHA* 1981; **11**: 11–30.

[2] Gray, S.D., Hirano, M. and Stato, K. Molecular and cellular structure of vocal fold tissue. In: Titze, I.R. (Ed.) *Vocal Fold Physiology: Frontiers of Basic Science*. 1st edn. San Diego, CA: Singular Publishing, 1993, pp. 1–34.

[3] Gray, S. Basmement membrane zone injury in vocal nodules. In: Gauffin, J. and Hammarberg, B. (Eds.) *Vocal Fold Physiology*. Singular Press, 1991.

[4] Hirano, M. Surgical anatomy and physiology of the vocal folds. In: Gould, W.J., Sataloff, R.T. and Spiegel, J.R. (Eds.) *Voice Surgery*. Chicago, IL: Mosby Year Book, 1993, pp. 125–158.

[5] Gray, S.D., Hammond, E. and Hanson, D.F. Benign pathologic responses of the larynx. *Ann. Otol. Rhinol. Laryngol.* 1995; **104**: 13–18.

[6] Jiang, J.J. A methodological study of hemilaryngeal phonation and the measurement of vocal cord intraglottal pressure and impact stress. Ph.D. thesis submitted to University of Iowa graduate college of Speech Pathology and Audiology, August, 1991

[7] Sataloff, R.T. The professional voice. In: Cummings, C.W., Fredrickson, J.M., Harker, L.A., Krause, C.J. and Schuller, D.E. (Eds.) *Otolaryngology-Head and Neck Surgery*. Vol. 3. St. Louis, MO: Mosby, 1986, pp. 2029–2056.

[8] Hirano, M. *et al.* Improved surgical technique for epidermoid cysts of the vocal fold. *Ann. Otol. Rhino. Laryngol.* 1989; **98**: 791–795.

[9] Courey, M.S. *et al.* Endoscopic vocal fold microflap: a three-year experience. *Ann. Otol. Rhinol. Laryngol.* 1995; **104**: 267–273.

[10] Courey, M.S., Garrett, C.G. and Ossoff, R.H. Medial microflap for excision of benign vocal fold lesions. *Laryngoscope* 1997; **107**: 340–344.

[11] Postma, G.N., Courey, M.S. and Ossoff, R.H. Microvascular lesions of the true vocal fold. *Ann. Otol. Rhino. Laryngol.* 1998; **107**: 472–476.

[12] Hochman, I. *et al.* Ectasias and varices of the vocal fold: clearing the striking zone. *Ann. Otol. Rhinol. Laryngol.* 1999; **108**: 10–16.

[13] Yanagihara, N. Significance of harmonic changes and noise components in hoarseness. *J. Speech Hear. Res.* 1967; **10**: 531–541.

[14] Gray, S.D. *et al.* Experimental approaches to vocal fold alteration: introduction to the minithyrotomy. *Ann. Otol. Rhinol. Laryngol.* 1999; **108**: 1–9.

[15] Hammond, T. *et al.* The intermediate layer: a morphologic study of the elastin and hyaluronic acid constituents of normal human vocal folds. *J. Voice.* 1997; **11**: 59–66.

[16] Titze, I.R. The physics of small-amplitude oscillation of the vocal folds. *J. Acoust. Soc. Am.* 1988; **83**: 1536–1552.

[17] Chan, R.W. and Titze, I.R. Viscosities of injectable biomaterials in vocal fold augmentation surgery. *Laryngoscope* 1998; **108**: 725–731.

[18] Archer, S.M. and Banks, E.R. Intracordal injection of autologous fat for augmentation of the mucosally damaged canine vocal fold: a long term histologic study. Presented at the 2nd World Congress on Laryngeal Cancer. Sydney, Australia, February 24, 1994.

